# Supplementary material for: A Peculiar Binding Characterization of DNA (RNA) Nucleobases at MoOS-Based Janus Biosensor: Dissimilar Facets Role on Selectivity and Sensitivity
Source: Biosensors (Basel). 2022 Jun 23;12(7):442. doi: 10.3390/bios12070442 (PMC9313196; doi:10.3390/bios12070442)
Supplement: Supplementary file 1 [file biosensors-12-00442-s001.zip › biosensors-1755476-supplementary.pdf]

biosensors

MDPI

Supplementary Materials

A Peculiar Binding Characterization of DNA (RNA) Nucleobases at MoOS-Based Janus Biosensor: Dissimilar Facets Role on Selectivity and Sensitivity

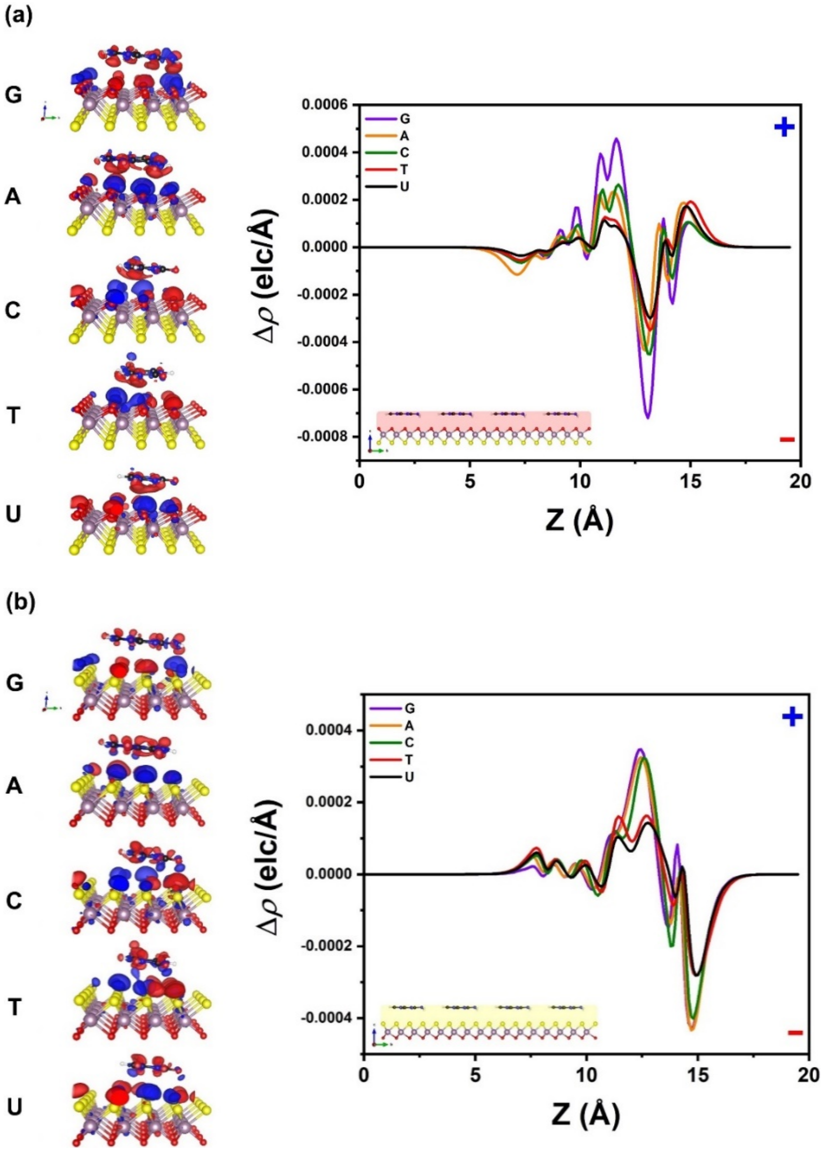

Figure S1. 3D–electronic density difference and profile of DNA (RNA) on (a) O–layer, (b) S–layer of the Janus MoOS monolayer, respectively. The red and blue colors illustrate isosurface electron accumulation and depletion ($\rho = \pm 3.5\times10^{-4}$ ē/Å^3), respectively.

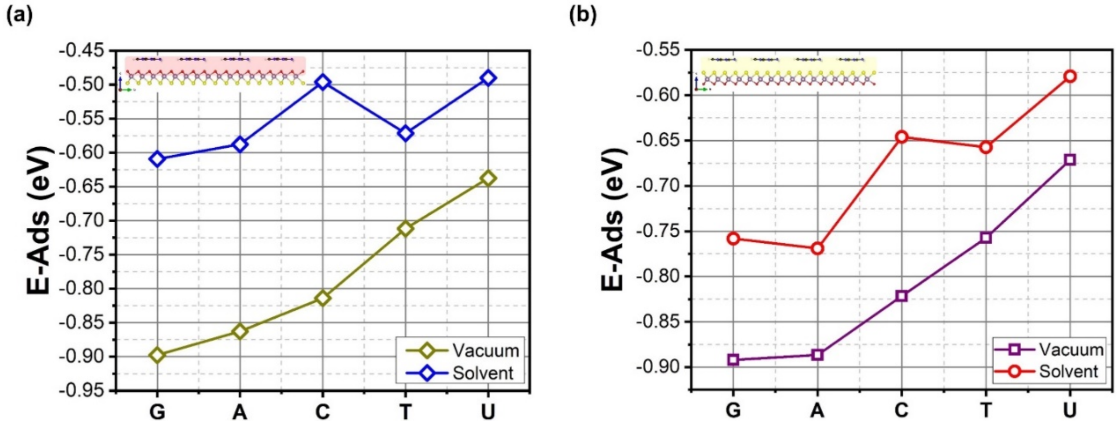

Figure S2. Comparison of the optimized adsorption energy at vacuum and implicit solvent model of the nucleobases molecules on the Janus MoOS monolayer facets, (a) on O–layer, (b) on S–layer, respectively.

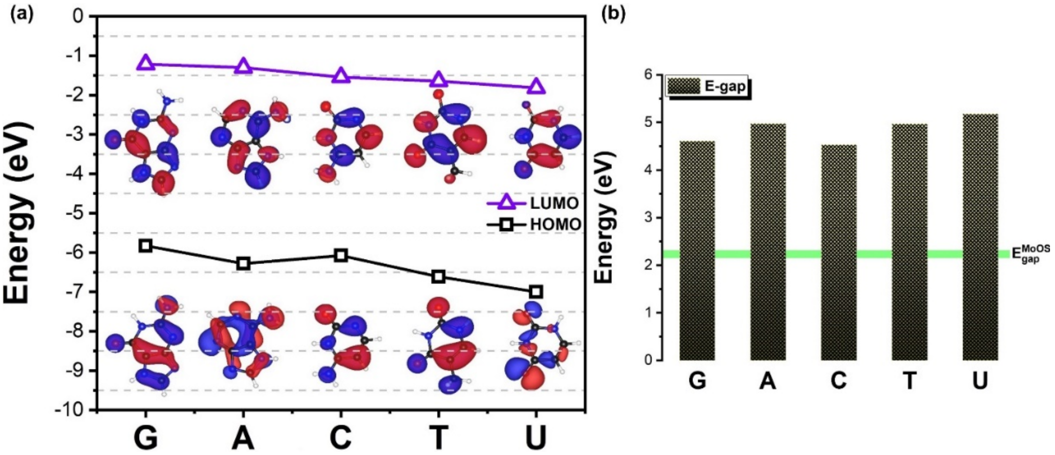

Figure S3. Spatial 3D–plots of the highest occupied (HOMO) and lowest unoccupied (LUMO) molecular orbitals and energy gap of nucleobases free molecules, respectively. The green bare illustrate the HOMO–LUMO gap (E^{gap} = 2.2eV) of the Janus MoOS monolayer. The red and blue colors illustrate isosurface electron accumulation and depletion (ρ = ±2.1×10^{-4} ē/$Å^3$), respectively.

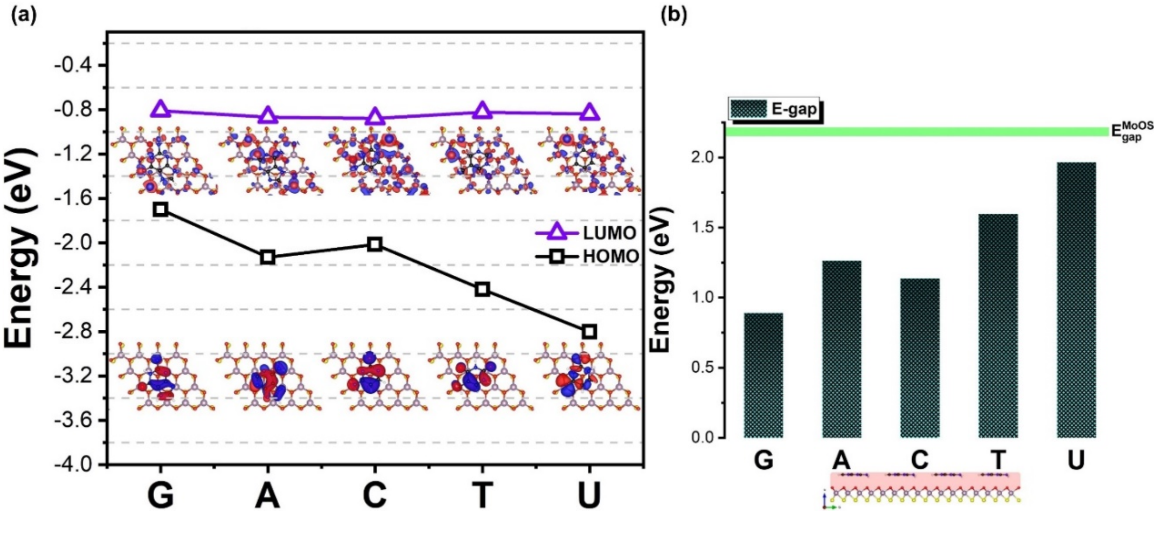

Figure S4. Spatial 3D–plots of the highest occupied (HOMO) and lowest unoccupied (LUMO) molecular orbitals and energy gap of nucleobases on O–layer of the Janus MoOS monolayer, respectively. The green bare illustrate the HOMO–LUMO gap (E^{gap} = 2.2eV) of the Janus MoOS monolayer. The red and blue colors illustrate isosurface electron accumulation and depletion (ρ = ±1.0×10^{-4} ē/Å^3), respectively.

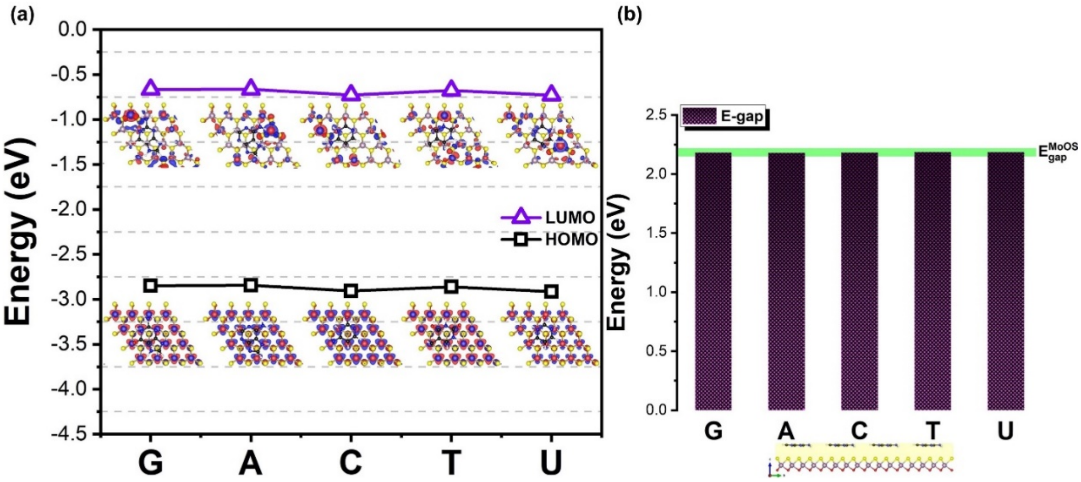

Figure S5. Spatial 3D–plots of the highest occupied (HOMO) and lowest unoccupied (LUMO) molecular orbitals and energy gap of nucleobases on S–layer of the Janus MoOS monolayer, respectively. The green bare illustrate the HOMO–LUMO gap (E^{gap} = 2.2eV) of the Janus MoOS monolayer. The red and blue colors illustrate isosurface electron accumulation and depletion (ρ = ±1.5×10^{-4} ē/Å^3), respectively.

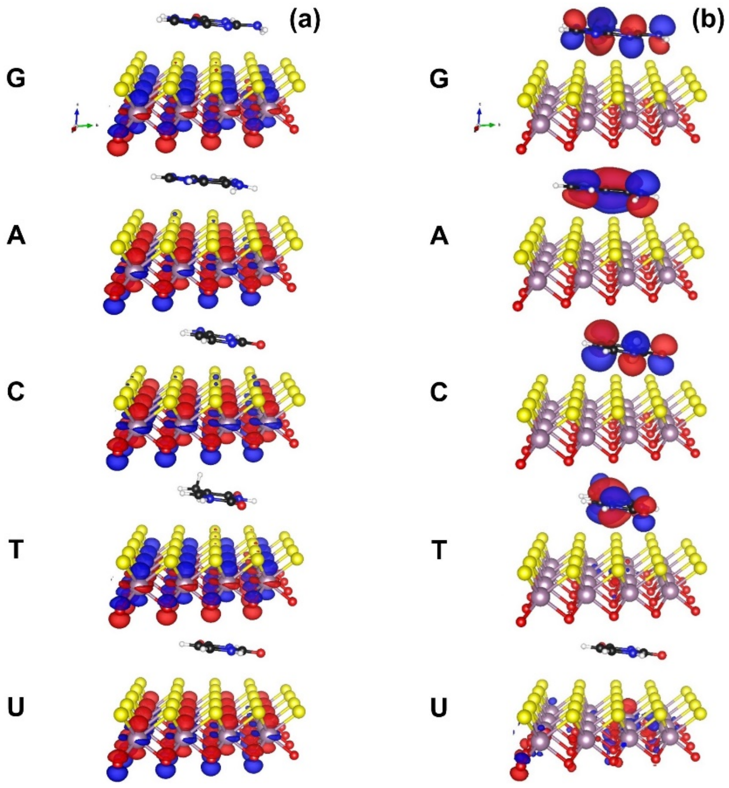

Figure S6. Spatial 3D–plots of (a) the highest occupied molecular orbitals (HOMO), (b) minus one (HOMO–1) molecular orbitals of DNA (RNA) on S–layer of the Janus MoOS monolayer. The red and blue colors illustrate isosurface electron accumulation and depletion ($\rho = \pm 1.5\times10^{-4}$ ē/Å^3), respectively.
